# Uncovering the health divide: examining the influence of poverty risk and income inequality on health outcomes in over 300 Finnish municipalities

**DOI:** 10.1007/s10198-025-01780-9

**Published:** 2025-05-25

**Authors:** Saqib Amin

**Affiliations:** Department of Economics, Accounting and Finance, Oulu Business School, University of Oulu, P.O. Box 4600, Oulu, FIN-90014 Finland

**Keywords:** Risk of poverty, Income inequality, Health outcomes, Finnish municipalities, I10, I14, O15, R11

## Abstract

The relationship between poverty, income inequality, and health outcomes has been extensively explored in the literature, primarily focusing on cross-country comparisons. However, findings from within-country analyses have yielded inconsistent results. This study investigates the association between poverty risk, income inequality, and health outcomes using Finnish municipality-level data from 1990 to 2023. Fixed-effect models are used to reveal a concerning interplay between these factors by utilizing measures of poverty risk, income inequality, and their synergistic effect, alongside a comprehensive set of health outcome indicators. The findings reveal a significant strong association between both poverty risk and income inequality with various health indicators. Moreover, the analysis also demonstrates a strong combined influence of poverty and inequality, indicating that their combined effect on negative health outcomes is more pronounced. These findings suggest that policies promoting social mobility and reducing income inequality may lead to a healthier Finnish population, particularly low-income residents, with a lower burden of chronic diseases and mortality.

## Introduction

Good health serves as a cornerstone of individual well-being and a critical determinant of societal prosperity.^1^ However, the distribution of health outcomes across populations is often uneven, with socioeconomic factors exerting a significant influence [23, 32, 33]. Despite advancements in healthcare, a persistent health divide exists, where individuals and communities facing economic hardship experience poorer health outcomes [2, 31]. This disparity manifests in various ways, ranging from elevated rates of chronic diseases (e.g., diabetes, heart disease) to increased prevalence of mental health problems and higher mortality rates [14, 19]. Understanding the underlying mechanisms driving this inequity is crucial for developing effective public health policies that promote health equity. This paper delves into the intricate relationship between income inequality and poverty risk, and their association with a spectrum of health indicators in Finnish municipalities.

While prior research has established associations between socioeconomic factors and health outcomes, findings often exhibit heterogeneity [25, 26]. Wilkinson and Pickett [44] in their book “The Spirit Level” highlighted the link between income inequality and health disparities and argued that income inequality has pervasive negative effects on various aspects of society. While influential, their work focused on cross-country comparisons and neglected poverty as a factor. This study incorporates a risk of poverty to control for the influence of inequality and explore their interaction. Thus, poverty emerges as a primary explanation for health and social issues in states where it is prevalent, overshadowing the role of inequality [22].

Finland presents a fascinating paradox. Consistently ranked among the World Happiness Countries for seven years (UN World Happiness Reports), it boasts a robust social safety net and welfare system. Yet, this seemingly advantageous social context coexists with a persistent health divide [27, 30]. Furthermore, Finland faces noteworthy socio-economic challenges despite its positive well-being ranking. These challenges include rising income inequality, elevated poverty rates, growing unemployment, and a concerning prevalence of mental health issues across municipalities [45]. In Finland, the at-risk-of-poverty rate has risen steadily from 7.9% in 1995 to 13.4% in 2022, while the Gini coefficient, measuring income inequality, has fluctuated but remained consistently moderate, ranging from 22.2 to 29.1 over the same period. These data paint a troubling picture of socioeconomic conditions in Finland. The rising at-risk-of-poverty rate signifies an increasing number of individuals and households struggling to meet their basic needs. Furthermore, the persistently high Gini coefficient reflects substantial income disparities across the population, indicating significant challenges in achieving equitable distribution of resources and opportunities. Against this backdrop, this study seeks to explore a crucial question: is there a discernible relationship between poverty, income inequality, and health outcomes within the Finnish population?

**Fig. 1:**
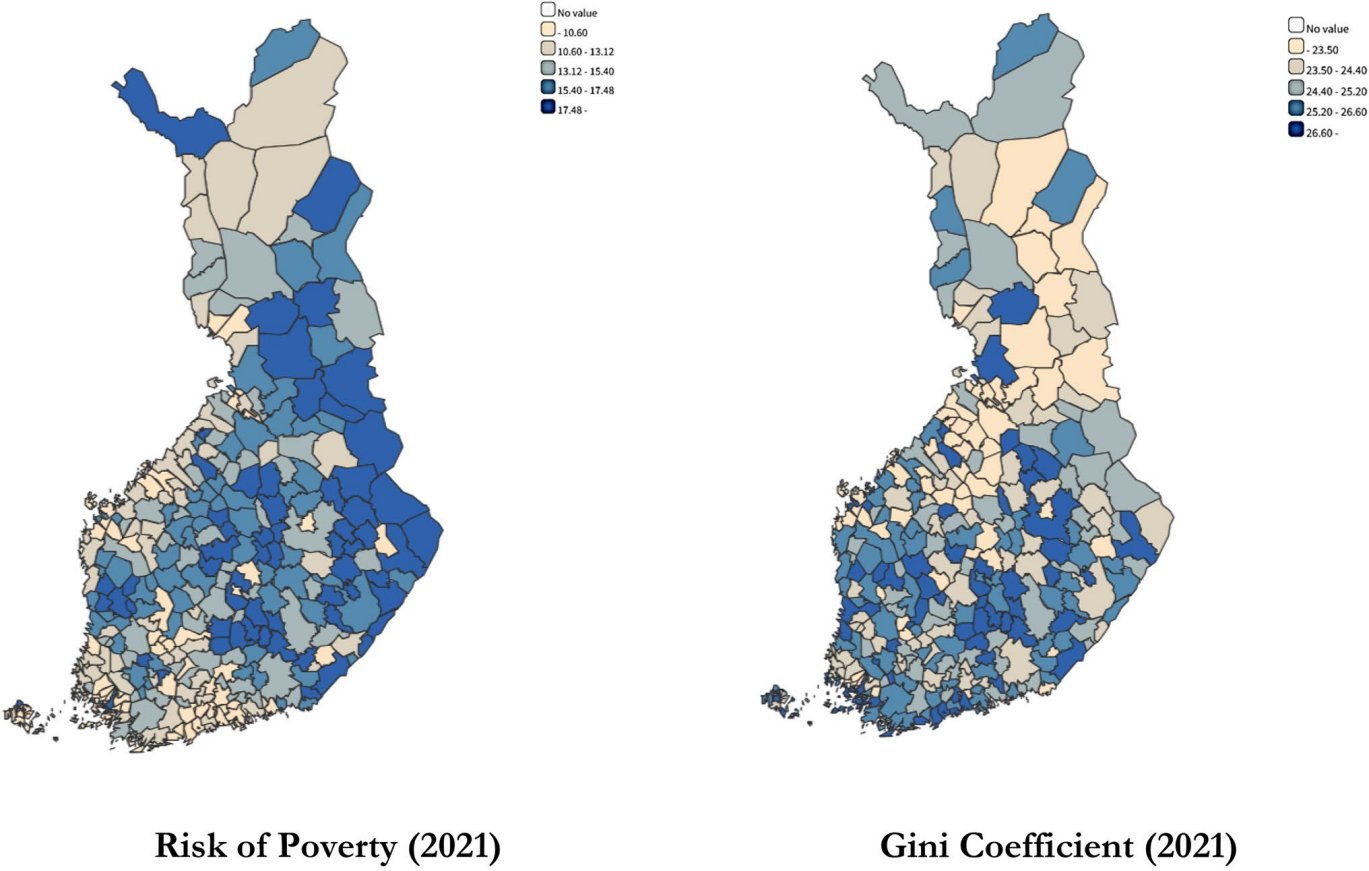
Mapping the current situation of risk of poverty (RPOV) and income equality (GINI) in Finnish Municipalities. Source: Sotkanet.fi, Statistical and indicator bank^©^ Institute of Health and Welfare

Figure 1 shows the situation worsens when considering regional differences among Finnish municipalities. According to the Finnish Institute of Health and Welfare (THL), Finnish municipalities exhibit substantial spatial heterogeneity in terms of poverty and income inequality. The at-risk-of-poverty rate demonstrates significant variation, ranging from a minimum of 2.9% to a maximum of 30.1%, highlighting stark disparities in economic well-being across different regions. Similarly, the Gini coefficient, a standard measure of income inequality, reveals considerable geographical variation, with values ranging from 15.9 to 66.1 (Statistical information on the health and well-being of the population, Sotkanet Statistics). This wide range underscores the multifaceted socio-economic landscape of Finland, where certain areas experience relatively low levels of both poverty and income inequality, while others face more pronounced socio-economic challenges.

It also highlights that in some municipalities, low at-risk-of-poverty rates coexist with high Gini coefficients, suggesting a scenario where wealth is concentrated among a smaller segment of the population, leading to greater income inequality within otherwise relatively wealthy areas. Conversely, other municipalities might exhibit high poverty rates coupled with relatively low Gini coefficients, implying a more evenly distributed, albeit lower overall level of economic well-being. This observed variability underscores the complexity of socio-economic dynamics within Finnish communities. In such a context, examining the combined effect of poverty and income inequality serves as a valuable proxy for understanding the socioeconomic disparities that may impact health outcomes in Finland. Analyzing these factors together offers deeper insights into the social and economic determinants of health disparities across regions. To address this gap, this study examines three key factors influencing health outcomes: (1) the direct effects of income inequality, (2) the direct role of poverty, and (3) the direct and synergistic effects of income inequality and poverty. It is hypothesized that the negative health impact of poverty risk is exacerbated in societies with greater income inequality. This synergistic effect suggests that these socioeconomic factors reinforce each other’s detrimental influence, leading to a more pronounced health divide.

By examining the complex interplay between these factors in Finland, this study aims to shed light on the underlying drivers of the health divide. The findings may inform the development of evidence-based policies aimed at promoting social mobility, reducing income inequality, and ultimately creating a healthier future for all Finnish citizens. A rich dataset encompassing over 300 Finnish municipalities over 33 years from 1990 to 2023 is used, allowing for the exploration of potential secular trends in health and socioeconomic disparities. By employing fixed-effect models, the analysis isolates the effects of poverty risk and income inequality on various health indicators, controlling for baseline differences between municipalities.

## Linkage between poverty–inequality–health nexus

A substantial body of research establishes a clear link between poverty and poor health outcomes [35, 42]. Poverty restricts access to essential resources like nutritious food, clean water, and adequate sanitation, increasing vulnerability to illness [20]. Do and Finch [11] emphasize the role of neighborhood context, where poverty can create environments with higher exposure to environmental hazards and fewer opportunities for healthy living. This creates a vicious cycle, as Thiede and Traub [39] point out, that poor health can further exacerbate poverty due to lost productivity and increased healthcare expenses.

Breaking the poverty-health cycle requires multifaceted interventions. Dartanto and Nurkholis [9] highlight the importance of identifying the dynamics of poverty, including factors like access to education and social safety nets. Studies like Tafran, Tumin, and Osman [37] show that poverty reduction strategies targeting income and employment can lead to improvements in life expectancy. Chapman and Hariharan [8] further emphasize the importance of addressing income inequality, as the health benefits of poverty reduction may be greater for those facing the most extreme deprivation.

Income inequality, a broader concept than poverty, also significantly influences health outcomes. Research suggests that higher levels of income inequality within a society are associated with worse population health [33]. Asafu‐Adjaye [4] finds a consistent negative association between income inequality and health status across various countries. Several mechanisms may explain this association. Kawachi and Kennedy [25] propose that income inequality can lead to underinvestment in social goods, disrupt social cohesion, and create negative psychological effects due to social comparisons.

The relationship between poverty, income inequality, and health is not always straightforward. Studies by Fiscella and Franks [16] and Latif [29] highlight the importance of individual income, suggesting that health disparities may persist even within high-income inequality settings. Biggs et al. [6] further emphasize the role of context, demonstrating that the impact of income inequality on health can vary depending on factors like national wealth and poverty trends [18, 24]. For example, research by Wilkinson and Pickett [44] in their influential book “The Spirit Level” argues that more equal societies tend to have better health outcomes for all citizens, regardless of their socioeconomic status. This suggests that income inequality creates a broader societal context that negatively impacts health.

Understanding these complexities is crucial for developing effective interventions. Efforts to reduce poverty, promote income equality, and address social determinants of health are essential for creating a society where everyone has the opportunity to achieve good health. For instance, Wen et al. [43] suggest that neighborhood affluence, rather than poverty or income inequality, maybe a more significant determinant of individual health in the United States. Similarly, Bakkeli [5] finds that individual characteristics play a more significant role in determining health risks in China, with income inequality having minimal influence. Furthermore, research delves into the mechanisms underlying these relationships. Diez-Roux, Link, and Northridge [10] find that income inequality in the US is linked to increased levels of cardiovascular disease risk factors, particularly among individuals with lower incomes. Similarly, Urakawa, Wang, and Alam [40] explore the role of time poverty, highlighting how a lack of time can hinder healthy behaviors in Japan.

While poverty and inequality are often used interchangeably, it represents distinct, yet intricately related phenomena. Income inequality acts as a breeding ground for poverty, with a wider income gap leading to a higher concentration of individuals falling below the poverty line [36]. However, poverty is not simply the extreme end of the income distribution. The Supplementary Benefit Commission in Britain defines poverty as a state of deprivation that leads to social exclusion and isolation (1979). This definition highlights a key point of interaction: poverty arises not just from low income, but from the social consequences of that low income. Limited financial resources can restrict access to education, healthcare, and opportunities for social participation, thereby reinforcing and deepening the cycle of poverty [7].

**Fig. 2:**
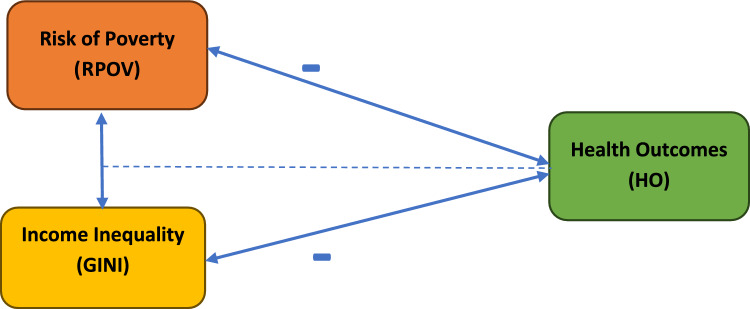
The relationship between the risk of poverty, income inequality, and health outcomes. Source: Author’s design

Figure 2 represents that poverty and inequality, while separate concepts, work together to worsen health outcomes. A wider income gap (inequality) creates more poverty, leading to limited resources and social exclusion. This social exclusion, marked by restricted access to education, healthcare, and social participation, reinforces the cycle of poverty. The negative effects extend beyond income, as residing in poverty-stricken communities creates a web of disadvantages. Limited access to healthy resources and social support networks due to poverty and social exclusion leads to chronic stress and poorer health. By recognizing this synergy between poverty and inequality, more effective health interventions can be developed. Policies aimed at reducing both poverty and inequality, along with investments in universal healthcare, education, and social safety nets, can create a fairer environment and empower individuals to make healthier choices. Ultimately, addressing the root causes of poverty, such as unemployment and lack of education, is essential for achieving long-term improvements in population health.

Further research is needed to explore the causal mechanisms linking poverty, income inequality, and health. Longitudinal studies and in-depth analyses of regional variations can provide valuable insights [17]. Additionally, investigating the effectiveness of various policy interventions aimed at reducing poverty and income inequality is crucial for improving population health outcomes [20].

This paper acknowledges the intricate relationship between poverty and inequality with health outcomes at Finnish municipality level. While they are undeniably interconnected, it is crucial to recognize their distinct characteristics. This distinction necessitates the utilization of comprehensive measures for both poverty and inequality to achieve a more nuanced understanding of how socioeconomic factors influence health outcomes. One promising approach to formally investigate this dynamic involves analyzing the statistical interactions between these measures. To our knowledge, such an exploration has been absent in existing literature and warrants further investigation, demanding careful scrutiny of underlying assumptions.

### Theoretical framework

In Finland, as in many countries, a significant health divide exists between different populations [12, 41]. Understanding the factors contributing to this gap is crucial for improving public health. This paper examines how social determinants of health (SDOH) and income inequality influence health outcomes across Finnish municipalities. SDOH theory posits that factors beyond individual choices and access to healthcare significantly impact health [13, 15]. Socioeconomic status, social support networks, and neighborhood environment all play a role. For example, individuals with lower socioeconomic status, often indicated by poverty risk, tend to have poorer health outcomes. Strong social connections, on the other hand, can buffer stress and promote healthy behaviors [1].

On a broader scale, the stress theory attempts to elucidate the general mechanisms linking income inequality and health. The stress theory posits that individuals continually compare themselves with others, with lower status often leading to feelings of inferiority, increased stress, and compromised health [3]. This theory suggests that in affluent countries, income inequality induces stress that adversely affects mental health and limits access to social support networks. Building on stress theory, Wilkinson and Pickett [44] theory, The Spirit Level focuses specifically on income inequality within a society. Their research suggests a strong correlation between income inequality and a range of health and social problems, even in wealthy countries. The theory argues that high levels of income inequality create a stressful social environment for everyone, not just those at the bottom. This stress can lead to poorer health outcomes across all income levels, along with increased rates of mental illness, violence, and various other diseases.

This theoretical framework underpinning the relationship between poverty, inequality, and health draws from two key theoretical perspectives: social determinants of health theory and Spirit level theory of inequality. Social determinants of health theory posits that disparities in health outcomes stem from unequal distribution of resources, opportunities, and social structures within society [21]. The Spirit Level theory of inequality argues that greater income inequality within countries is linked to a wide range of social and health problems, including higher levels of violence, mental illness, drug abuse, obesity, and poor educational achievement [44]. Individuals with higher socioeconomic status generally enjoy better access to healthcare, education, and healthier living conditions, leading to improved health outcomes. Conversely, individuals facing socioeconomic disadvantages encounter barriers to accessing essential resources, resulting in poorer health outcomes.

By analyzing these data, this study aims to demonstrate the connection between poverty risk, income inequality, and health outcomes. It is expected that higher income inequality and poverty risk correlate with poorer health outcomes, even in wealthier municipalities. This combined framework, integrating SDOH and The Spirit Level, provides a robust foundation for understanding the relationship between social inequality and health outcomes. It highlights how the unequal distribution of resources, opportunities, and social structures within society (as emphasized by SDOH theory) combined with the broader social stress caused by high-income inequality (as emphasized by The Spirit Level) contribute to health disparities.

This synthesis of SDOH and The Spirit Level perspectives supports a comprehensive understanding of the multifaceted relationship between social inequality and health outcomes. The analysis focuses on data from 300 Finnish municipalities spanning the period from 1990 to 2023. By examining this dataset, the study seeks to clarify how poverty risk and income inequality interact to shape population health.

## Description of statistical methods and data sources

### Data

nnual data spanning from 1990 to 2023 were gathered from SOTKAnet (www.sotkanet.fi), a comprehensive repository of municipal-level statistical data on welfare and health in Finland. Ten health outcome indicators were employed from SOTKAnet, including the mental health index, cancer index, coronary disease index, accidental injury index, cerebrovascular disease index, musculoskeletal disorder index, Kela’s mortality index, Kela’s disability index, Thl’s morbidity index, and mortality rate (see Table 1). To assess socioeconomic factors, data on the Gini coefficient (income inequality) and the at-risk-of-poverty rate were utilized. The Gini coefficient, retrieved from the income distribution register within SOTKAnet, reflects the distribution of disposable income among households within each municipality. Ranging from 0 to 1 (with higher values indicating greater inequality), the coefficient was multiplied by 100 for analysis. The at-risk-of-poverty rate, on the other hand, identifies the proportion of individuals living in households with incomes below the national at-risk-of-poverty threshold (60% of the median equivalent disposable income). This metric captures the low-income population within each municipality relative to the national standard. In addition, several control variables were included in the empirical analysis; such as educational level, unemployment rate, social assistance recipients, family structure, economic dependency ratio, net migration, and municipal expenditure.

**Table 1:** Description of the variables used and their expected signs in the regression

Variable type	Symbol	Variable description/definition	Database used
Dependent variables: health outcomes indicators			
Mental health index	MHI	MHI is comprised of three parts (1) Suicides and suicide attempts leading to hospitalization; (2) Entitlement to special refunds for psychosis-related medication; (3) Disability pensions due to mental health issues	Finnish National Institute for Health and Welfare (THL) https://sotkanet.fi/sotkanet/en/index
Cancer index	CI	CI tracks new cancer cases among individuals aged 0–79 years, compared to the prevalence in the total population. It includes all cancer diagnoses except non-melanoma skin cancers	
Coronary disease index	CDI	CDI measures coronary disease prevalence among 35–79-year-olds, including heart attacks, angina, and related causes of death, compared to the total population	
Accidental injury index	AII	AII measures hospitalizations for injuries, poisonings, and external causes among individuals aged 15–79 years, comparing it to the total population	
Cerebrovascular disease index	CVDI	CDI tracks the prevalence of first attacks of cerebrovascular disease resulting in death or hospitalization among individuals aged 35–79 years, compared to the total population	
Musculoskeletal disorder index	MDI	MDI measures the prevalence of musculoskeletal disorders among individuals aged 16–64 years as represented by disability pensions due to musculoskeletal conditions. It is reported as an index, where higher values indicate a greater prevalence of musculoskeletal disorders in the municipality	
Kela’s mortality index	KMI	KMI measures the number of deaths as a proportion of the total population in each Finnish municipality	
Kela’s disability index	KDI	MDI measures the number of disability pension recipients as a proportion of the total working-age population (16–64 year-olds) in each Finnish municipality	
Thl’s morbidity index	TMI	TMI evaluates local morbidity relative to the national average across seven disease groups, using four factors: mortality, disability, quality of life, and healthcare expenditure	
Mortality	MOR	MOR shows the number of deaths and death rate per 100,000 inhabitants by sex	
Main explanatory variables			
Risk of poverty	RPOV	RPOV measures the percentage of individuals in a geographic area living in low-income households relative to the total population. The poverty threshold is set at 60% of the median equivalent disposable income of Finnish households, adjusted using the OECD scale	
Gini coefficient	GINI	GINI displays how disposable income is distributed among household-dwelling units in the region, using the Gini coefficient adjusted by the consumption unit to account for household size and structure	
Control Variables			
Educational level	EDU	Average length of education and training for each individual’s highest educational qualification or degree after basic education, representing the level of education in the population	
Unemployed people	UN	The unemployed population as a percentage of the total labour force	
Social assistance	SA	Number of households receiving social assistance during the calendar year	
Families	FAM	Number of families with children are those with at least one child under 18	
Eco. dependency ratio	EDR	Number of economically inactive individuals, including the unemployed and those outside the labor force, per hundred employed individuals	
Net migration	MIG	Intermunicipal net migration per thousand inhabitants	
Municipal expenditure	MEXP	Per-capita operating net expenditure of municipal operational economy in euros	

### Method

This study employs fixed-effect regression models to analyze the relationship between poverty, income inequality, and health outcomes. Municipality-level fixed effect are included to control for time-invariant characteristics of each municipality (e.g., geographic location, cultural norms) [28, 38]. This approach enables the analysis to isolates the effects of poverty and income inequality on health outcomes while focusing on the variation within municipalities over time. The independent variables in analysis include the Gini coefficient (income inequality) and the risk of poverty within each municipality. Additionally, we incorporate several control variables, such as educational level, unemployment rate, and social assistance recipients, to account for other factors that might influence health outcomes. The dependent variable for each model is a specific health indicator, with various indicators examined throughout the study. The decision to utilize fixed-effect models stems from the substantial variation within municipalities in the main explanatory variables (poverty and inequality) and the dependent variables (health outcomes).

It is hypothesized that short-term changes in income inequality, measured by both the Gini coefficient and poverty risk, will be positively correlated with various health outcomes. To explore this hypothesis, three models were developed for each health indicator: Model 1: This model includes only the intercept and the municipal risk of poverty. Model 2: This model includes only the intercept and the municipal Gini coefficient. Model 3: This model incorporates the intercept, the municipal Gini coefficient, and the municipal risk of poverty (as an interaction term). This allows us to examine the independent and combined effects of poverty and income inequality on health outcomes. Following this initial analysis, all three models were run for each health indicator with the inclusion of all control variables. This allows us to assess the impact of poverty and inequality while accounting for the influence of other relevant factors.

Based on the theoretical framework, the standard model of Rambotti [34] is employed in the empirical findings. To assess the baseline relationship between poverty and health outcomes, a baseline model was first estimated without any control variables. The equation for the baseline model is as follows:

1 $${\text{HO}}_{i,t}=\alpha + {\theta }_{1}{RPOV}_{i,t}+ {\vartheta }_{2}{GINI}_{i,t}+ {\chi }_{3}{RPOV*GINI}_{i,t}+{\varphi }_{\text{t}}+ {\upmu }_{\text{i}}+{\in }_{i,t}$$

Subsequently, a series of increasingly complex models were estimated to examine the robustness of the findings and to account for potential confounding factors. These models incorporated a comprehensive set of control variables, including. The equation for the full model is as follows:

2 $${\text{HO}}_{i,t}=\alpha + {\theta }_{1}{RPOV}_{i,t}+ {\vartheta }_{2}{GINI}_{i,t}+ {\chi }_{3}{RPOV*GINI}_{i,t}+{\tau }_{4}{X}_{i,t} +{\varphi }_{\text{t}}+ {\upmu }_{\text{i}}+{\in }_{i,t}$$

where ${HO}_{i,t}$ is the health outcomes indicators, which include mental health index, cancer index coronary disease index accidental injury index cerebrovascular disease index musculoskeletal disorder index Kela’s mortality index Kela’s disability index Thl’s morbidity index and mortality for country *i* at the time *t*. ${RPOV}_{i,t}$ presents at the risk of poverty and ${GINI}_{i,t}$ shows the income inequality and ${RPOV*GINI}_{i,t}$ presents the interaction term of risk of poverty and income inequality. Finally, ${X}_{i,t}$ is the set of control variables including educational level, unemployed people, social assistance, families, economic dependency ratio, net migration and municipal expenditure. The model includes municipality fixed-effects (${\mu }_{i}$) to account for time-invariant unobserved heterogeneity across municipalities, and time fixed-effects (${\varphi }_{t}$) to control for factors that vary over time but are constant across municipalities. This two-way fixed-effects model controls for both municipality-level and time-specific confounders.

## Empirical findings

This study investigates the relationship between poverty risk (measured by the risk of poverty), income inequality (represented by the Gini coefficient), and various health indicators across Finnish municipalities. Descriptive statistics are provided in Table 2, offering an initial overview of the data. Table 3 presents the outcomes of robust relationships between poverty risk, income inequality, and their interaction term (synergy) on various health indicators, excluding control variables. Tables 4, 5, and 6 incorporate control variables in each model to examine the independent and combined effects of poverty risk, income inequality, and their interaction (synergy) on health outcomes.

**Table 2:** Descriptive statistics

Variables	Mean	Median	Maximum	Minimum	SD	Sum	Observations
MHI	111.4757	106.7	268.2	10.6	35.42054	598,736.2	5371
CI	94.2592	94.2	160.5	47.1	13.10345	447,919.7	4752
CDI	139.098	131.65	336.3	21.3	45.69908	660,993.6	4752
AII	127.0353	124.4	223.9	43.5	24.59296	603,671.6	4752
CVDI	121.7941	118.5	271.9	21.2	28.93008	578,765.8	4752
MDI	180.724	173.7	398.1	18.4	59.62215	858,800.6	4752
KMI	101.812	101.6	201.6	18.9	13.61211	880,877.1	8652
KDI	111.1833	108.7	241.9	0	29.67589	961,958.3	8652
TMI	126.6225	124.55	237.9	19.6	27.23908	704,274.5	5562
MOR	1197.027	1153.6	3883.5	0	402.4248	12,206,088	10,197
RPOV	14.0974	14.2	30.1	2.9	4.451583	121,829.7	8642
GINI	24.45625	24.2	66.1	15.9	2.856312	211,595.5	8652
GINI x RPOV	346.1026	342.24	1312.36	67.86	120.2565	2,991,019	8642
EDU	255.757	250.2	603.9	114.1	62.95271	2,607,954	10,197
UN	12.11569	11.4	33.9	0	5.331713	122,259.4	10,091
SA	907.1347	237	52,539	5	2909.857	8,811,906	9714
FAM	4629.691	1781.5	161,417	21	11,193.68	45,778,387	9888
EDR	161.5112	158.6	304.8	72.9	34.71005	1,646,930	10,197
MIG	−3.56448	−3.7	84.7	−99.1	9.62097	−36,347	10,197
MEXP	4396.617	4187.35	10,621.9	0	1519.543	38,039,527	8652

**Table 3:** Fixed effect (FE) estimates of risk of poverty (RPOV) and income inequality (GINI) on health outcomes (without control variables)

Variables	(1)	(2)	(3)	(4)	(5)	(6)	(7)	(8)	(9)	(10)
	MHI	CI	CDI	AII	CVDI	MDI	KMI	KDI	TMI	MOR
Model-I										
RPOV	2.113^†††^	−0.205	2.071^†††^	0.045	0.630^†^	1.218^†††^	0.195^††^	0.690^†††^	0.861^†††^	11.92^†††^
	(0.210)	(0.161)	(0.340)	(0.195)	(0.329)	(0.258)	(0.087)	(0.067)	(0.098)	(1.784)
C	80.62^†††^	97.28^†††^	108.6^†††^	126.3^†††^	112.5^†††^	162.8^†††^	99.28^†††^	102.7^†††^	113.8^†††^	1040.9^†††^
	(3.073)	(2.382)	(5.013)	(2.877)	(4.840)	(3.801)	(1.243)	(0.964)	(1.479)	(25.27)
Obs	5371	4752	4752	4752	4752	4752	7097	7097	5557	8642
Cross	297	264	264	264	264	264	309	309	309	309
* R*-squared	0.840	0.408	0.784	0.754	0.498	0.927	0.503	0.939	0.919	0.708
Time FE	Yes	Yes	Yes	Yes	Yes	Yes	Yes	Yes	Yes	Yes
Year FE	Yes	Yes	Yes	Yes	Yes	Yes	Yes	Yes	Yes	Yes
Model-II										
GINI	0.469^††^	−0.144	0.040	0.516^†††^	0.126	0.464^††^	0.281^†††^	−0.036	0.375^†††^	3.730^††^
	(0.184)	(0.141)	(0.298)	(0.170)	(0.286)	(0.225)	(0.085)	(0.066)	(0.084)	(1.750)
C	99.81^†††^	97.84^†††^	138.0^†††^	114.1^†††^	118.6^†††^	169.1^†††^	108.8^†††^	113.2	117.2^†††^	1117.4^†††^
	(4.597)	(3.513)	(7.424)	(4.239)	(7.140)	(5.617)	(2.093)	(1.626)	(2.117)	(42.88)
Obs	5371	4752	4752	4752	4752	4752	7107	7107	5562	8652
Cross	297	264	264	264	264	264	309	309	309	309
* R*-squared	0.837	0.408	0.782	0.755	0.498	0.926	0.496	0.936	0.918	0.700
Time FE	Yes	Yes	Yes	Yes	Yes	Yes	Yes	Yes	Yes	Yes
Year FE	Yes	Yes	Yes	Yes	Yes	Yes	Yes	Yes	Yes	Yes
Model-III										
RPOV	2.992^†††^	−0.481	3.410^††^	0.096	4.402^†††^	1.356	0.348	0.457^†^	0.717^†^	20.16^††^
	(0.846)	(0.636)	(1.339)	(0.768)	(1.291)	(1.014)	(0.357)	(0.277)	(0.418)	(7.171)
GINI	0.756^††^	−0.283	0.576	0.472^†^	2.611^†††^	0.987^†^	0.464^††^	−0.097	0.219	6.356^†^
	(0.473)	(0.358)	(0.753)	(0.432)	(0.726)	(0.571)	(0.190)	(0.147)	(0.232)	(3.870)
RPOV × GINI	0.035^†††^	0.011^†^	0.051^††^	0.003^††^	0.195^†††^	0.098^†††^	0.022^††^	0.009^†††^	0.004^†††^	0.328^†††^
	(0.031)	(0.023)	(0.050)	(0.028)	(0.048)	(0.038)	(0.013)	(0.010)	(0.015)	(0.268)
C	61.89^†††^	104.2^†††^	93.48^†††^	115.5^†††^	180.1^†††^	189.2^†††^	110.6^†††^	105.2^†††^	108.9^†††^	883.1^†††^
	(12.37)	(9.415)	(19.81)	(11.36)	(19.09)	(15.01)	(4.912)	(3.812)	(6.114)	(101.0)
Obs	5371	4752	4752	4752	4752	4752	7097	7097	5557	8642
Cross	297	264	264	264	264	264	309	309	309	309
* R*-squared	0.840	0.408	0.784	0.755	0.500	0.927	0.504	0.939	0.919	0.708
Time FE	Yes	Yes	Yes	Yes	Yes	Yes	Yes	Yes	Yes	Yes
Year FE	Yes	Yes	Yes	Yes	Yes	Yes	Yes	Yes	Yes	Yes

Robust standard errors in parentheses, ^†††^*p* < 0.01, ^††^*p* < 0.05, ^†^*p* < 0.10

**Table 4:** Fixed effect (FE) estimates of the risk of poverty (RPOV) on health outcomes (Model-I)

Variables	(1)	(2)	(3)	(4)	(5)	(6)	(7)	(8)	(9)	(10)
	MHI	CI	CDI	AII	CVDI	MDI	KMI	KDI	TMI	MOR
RPOV	1.404^†††^	−0.221	1.218^†††^	0.099	0.404^††^	0.321^†††^	0.120^††^	0.283^†††^	0.721^†††^	8.160^†††^
	(0.245)	(0.183)	(0.384)	(0.220)	(0.372)	(0.289)	(0.086)	(0.068)	(0.107)	(1.799)
EDU	−0.248^†††^	0.010	0.093	−0.118^†††^	−0.096	−0.162^†††^	−0.016	−0.191^†††^	−0.094^†††^	−3.030^†††^
	(0.046)	(0.035)	(0.073)	(0.042)	(0.071)	(0.055)	(0.017)	(0.013)	(0.021)	(0.345)
UN	0.089	0.185	0.887^††^	−0.323	0.061	0.829^†††^	0.749^†††^	0.997^†††^	0.332^†††^	12.75^†††^
	(0.225)	(0.172)	(0.361)	(0.207)	(0.350)	(0.272)	(0.091)	(0.0729)	(0.105)	(1.821)
SA	0.0007	0.0005	0.001	−0.0003	−5.29E	0.0006	−0.0001	0.0005^†††^	0.0003	0.003
	(0.0006)	(0.0004)	(0.0009)	(0.0005)	(0.0009)	(0.0007)	(0.0002)	(0.0001)	(0.0003)	(0.004)
FAM	−0.0005	−0.0007^†^	−0.0002	0.0006	0.0003	−0.001^††^	−0.0003^††^	−0.0009^†††^	0.0002	−0.005^†^
	(0.0005)	(0.0004)	(0.0008)	(0.0004)	(0.0008)	(0.0006)	(0.0001)	(0.0001)	(0.0002)	(0.003)
EDR	0.142^†††^	0.003	0.126^†^	0.159^†††^	−0.001	0.179^†††^	0.093^†††^	0.223^†††^	0.108^†††^	0.328
	(0.040)	(0.031)	(0.065)	(0.037)	(0.063)	(0.049)	(0.015)	(0.012)	(0.018)	(0.316)
MIG	−0.0036	−0.021	0.023	−0.044	−0.014	−0.063	0.004	0.013	−0.039^†††^	−1.127^†††^
	(0.034)	(0.026)	(0.054)	(0.031)	(0.053)	(0.041)	(0.015)	(0.012)	(0.015)	(0.313)
MEXP	−5.30E	0.0005	−0.003^††^	−0.002^†††^	−0.001	−0.005^†††^	−0.0009^††^	−0.002^†††^	−0.0005	−0.153^†††^
	(0.0007)	(0.0006)	(0.001)	(0.0007)	(0.001)	(0.0009)	(0.0003)	(0.0003)	(0.0003)	(0.007)
C	143.3^†††^	92.61^†††^	82.12^†††^	54.17^†††^	146.0^†††^	223.4^†††^	108.1^†††^	126.1^†††^	130.5^†††^	1299.8^†††^
	(17.16)	(12.96)	(27.18)	(15.59)	(26.35)	(20.48)	(6.258)	(4.961)	(7.780)	(127.7)
Obs	5075	4749	4749	4749	4749	4749	6970	6970	5451	7880
Cross	297	264	264	264	264	264	308	308	308	308
*R*-squared	0.846	0.408	0.786	0.756	0.498	0.928	0.562	0.947	0.922	0.7626
Time FE	Yes	Yes	Yes	Yes	Yes	Yes	Yes	Yes	Yes	Yes
Year FE	Yes	Yes	Yes	Yes	Yes	Yes	Yes	Yes	Yes	Yes

Robust standard errors in parentheses, ^†††^*p* < 0.01, ^††^*p* < 0.05, ^†^*p* < 0.10

**Table 5:** Fixed effect (FE) estimates of income inequality (GINI) on health outcomes (Model-II)

Variables	(1)	(2)	(3)	(4)	(5)	(6)	(7)	(8)	(9)	(10)
	MHI	CI	CDI	AII	CVDI	MDI	KMI	KDI	TMI	MOR
GINI	0.397^††^	−0.123	0.068	0.575^†††^	0.105	0.390^†^	0.121^†††^	−0.091	0.308^†††^	2.063^††^
	(0.192)	(0.141)	(0.297)	(0.170)	(0.288)	(0.223)	(0.078)	(0.062)	(0.084)	(1.667)
EDU	−0.299^†††^	0.018	0.043	−0.121^†††^	−0.111	−0.171^†††^	−0.025	−0.212^†††^	−0.125^†††^	−3.557^†††^
	(0.045)	(0.034)	(0.072)	(0.041)	(0.069)	(0.054)	(0.016)	(0.012)	(0.021)	(0.325)
UN	0.081	0.156	1.048^†††^	0.309	0.115	0.787^†††^	0.738^†††^	1.012^†††^	0.263^††^	13.05^†††^
	(0.224)	(0.170)	(0.358)	(0.205)	(0.346)	(0.269)	(0.091)	(0.072)	(0.105)	(1.822)
SA	0.0007	0.0005	0.001	−0.0003	−7.31E	0.0006	−0.0001	0.0005^†††^	0.0003	0.003
	(0.0006)	(0.0004)	(0.0009)	(0.0005)	(0.0009)	(0.0007)	(0.0002)	(0.0001)	(0.0003)	(0.004)
FAM	−0.0003	−0.0007^†^	−5.81E−	0.0006	0.0003	0.001^†^	−0.0002^††^	−0.0009^†††^	0.0003	−0.005
	(0.0005)	(0.0004)	(0.0008)	(0.0004)	(0.0008)	(0.0006)	(0.0001)	(0.0001)	(0.0002)	(0.003)
EDR	0.181^†††^	−0.002	0.155^††^	0.166^†††^	0.0094	0.191^†††^	0.093^†††^	0.227^†††^	0.126^†††^	0.466
	(0.040)	(0.030)	(0.064)	(0.037)	(0.062)	(0.048)	(0.015)	(0.012)	(0.018)	(0.316)
MIG	−0.013	−0.019	0.014	−0.044	−0.017	−0.065	0.002	0.009	−0.047^†††^	−1.033^†††^
	(0.034)	(0.026)	(0.054)	(0.031)	(0.053)	(0.041)	(0.015)	(0.012)	(0.015)	(0.312)
MEXP	−0.001	0.0007	−0.004^†††^	−0.002^†††^	−0.001	−0.006^†††^	−0.0008^††^	−0.002^†††^	−0.001^†††^	−0.148^†††^
	(0.0007)	(0.0005)	(0.001)	(0.0007)	(0.001)	(0.0009)	(0.0003)	(0.0003)	(0.0003)	(0.007)
C	164.6^†††^	90.91^†††^	109.6^†††^	39.58^†††^	152.6^†††^	219.7^†††^	115.4^†††^	133.3^†††^	140.8^†††^	1502.1^†††^
	(17.16)	(12.88)	(27.04)	(15.47)	(26.18)	(20.35)	(5.927)	(4.704)	(7.629)	(122.7)
Obs	5075	4749	4749	4749	4749	4749	6970	6970	5451	7880
Cross	297	264	264	264	264	264	308	308	307	308
*R*-squared	0.845	0.408	0.786	0.757	0.498	0.928	0.562	0.947	0.922	0.762
Time FE	Yes	Yes	Yes	Yes	Yes	Yes	Yes	Yes	Yes	Yes
Year FE	Yes	Yes	Yes	Yes	Yes	Yes	Yes	Yes	Yes	Yes

Robust standard errors in parentheses, ^†††^*p* < 0.01, ^††^*p* < 0.05, ^†^*p* < 0.10

**Table 6:** Fixed effect (FE) estimates of interaction term of risk of poverty (RPOV) and income inequality (GINI) on health outcomes (Model-III)

Variables	(1)	(2)	(3)	(4)	(5)	(6)	(7)	(8)	(9)	(10)
	MHI	CI	CDI	AII	CVDI	MDI	KMI	KDI	TMI	MOR
RPOV	0.987^††^	−0.675	1.892^†^	−0.725	4.911^†††^	2.751^†††^	0.141	0.622^††^	0.028	36.42^†††^
	(0.901)	(0.661)	(1.388)	(0.795)	(1.343)	(1.044)	(0.325)	(0.257)	(0.417)	(6.993)
GINI	0.083^††^	−0.353	0.323	0.179	2.711^†††^	1.219^††^	0.130	−0.379^†^	−0.100	15.40^†††^
	(0.495)	(0.362)	(0.761)	(0.436)	(0.736)	(0.572)	(0.174)	(0.138)	(0.229)	(3.766)
RPOV × GINI	0.014***	0.018^†^	0.025^††^	0.028	0.203^†††^	0.116^†††^	0.0002	0.035^†††^	0.025^††^	1.120^†††^
	(0.033)	(0.024)	(0.051)	(0.029)	(0.049)	(0.038)	(0.012)	(0.009)	(0.015)	(0.266)
EDU	−0.246^†††^	0.010	0.092	−0.122^†††^	−0.089	−0.156^†††^	−0.015^†††^	−0.189^†††^	−0.094^†††^	−3.098^†††^
	(0.046)	(0.035)	(0.073)	(0.042)	(0.071)	(0.055)	(0.017)	(0.013)	(0.021)	(0.346)
UN	−0.087	0.181	0.889	−0.315	0.043	0.834^†††^	0.746	1.018^†††^	0.327^†††^	12.05^†††^
	(0.225)	(0.172)	(0.361)	(0.207)	(0.349)	(0.272)	(0.092)	(0.073)	(0.105)	(1.826)
SA	0.0008	0.0005	0.001	−0.0002	0.0002	0.0008	−0.0001	0.0006^†††^	0.0003	0.0035
	(0.0006)	(0.0004)	(0.0009)	(0.0005)	(0.0009)	(0.0007)	(0.0002)	(0.0001)	(0.0003)	(0.004)
FAM	−0.0005	−0.0007^†^	−0.0002	0.0006	0.0001	0.001^††^	−0.0002^†^	−0.0009^†††^	0.0002	−0.005^†^
	(0.0005)	(0.0004)	(0.0008)	(0.0004)	(0.0008)	(0.0006)	(0.0001)	(0.0001)	(0.0002)	(0.003)
EDR	0.149^†††^	0.006	0.120^†^	0.172^†††^	0.040	0.207^†††^	−0.094^†††^	0.229^†††^	0.115^†††^	0.160
	(0.041)	(0.031)	(0.066)	(0.037)	(0.063)	(0.049)	(0.016)	(0.012)	(0.018)	(0.319)
MIG	−0.003	−0.020	0.023	−0.043	−0.009	−0.060	0.004	0.014	−0.039^†††^	1.093^†††^
	(0.034)	(0.026)	(0.054)	(0.031)	(0.053)	(0.041)	(0.015)	(0.012)	(0.015)	(0.312)
MEXP	−4.03E	0.0005	−0.003^††^	0.002^†††^	−0.0007	−0.005^†††^	0.0009^††^	0.002^†††^	−0.0005	0.153^†††^
	(0.0007)	(0.0006)	(0.001)	(0.0007)	(0.001)	(0.0009)	(0.0003)	(0.0003)	(0.0003)	(0.007)
C	140.59^†††^	101.0^†††^	74.79^††^	48.09^†††^	208.0^†††^	250.2^†††^	111.1^†††^	134.8^†††^	132.9^†††^	945.4^†††^
	(20.71)	(15.52)	(32.55)	(18.64)	(31.50)	(24.50)	(7.428)	(5.883)	(9.396)	(154.0)
Obs	5075	4749	4749	4749	4749	4749	6970	6970	5451	7880
Cross	297	264	264	264	264	264	308	308	307	308
*R*-squared	0.846	0.408	0.786	0.757	0.500	0.928	0.562	0.947	0.923	0.763
Time FE	Yes	Yes	Yes	Yes	Yes	Yes	Yes	Yes	Yes	Yes
Year FE	Yes	Yes	Yes	Yes	Yes	Yes	Yes	Yes	Yes	Yes

Robust standard errors in parentheses, ^†††^*p* < 0.01, ^††^*p* < 0.05, ^†^*p* < 0.10

Table 2 details descriptive statistics for the analysis variables, revealing their distribution and variability. Health outcomes have been examined through the various health-related indicators at the Finnish municipality level. The average mental health index (MHI) is 111.48, with a standard deviation of 35.42. This suggests that there is considerable variation in mental health across the municipalities. The cancer index (CI) ranges from 47.1 to 160.5, with a mean of 94.26 and a median of 94.2, while the Coronary Disease Index (CDI) (mean: 139.10, median: 131.65) shows more variation and the Musculoskeletal Disorder Index (MDI) shows a mean of 180.72 and a median of 173.7. The values of MDI range from 0 to 241.9, indicating variation in the extent of musculoskeletal health issues across different municipalities. Social inequality metrics like Risk of Poverty (RPOV) ranges from 2.9 to 30.1, with a mean of 14.10 and a median of 14.2. The GINI coefficient ranges from 15.9 to 66.1, with a mean of 24.46 and a median of 24.2. Lower values indicate more equal income distribution.

Table 3 explores how social inequality factors like poverty and income inequality influence health outcomes. It utilizes fixed effect (FE) models and presents results across three model specifications to offer a comprehensive picture. Furthermore, to account for potential spatial autocorrelation and heteroscedasticity, robust standard errors were employed in the fixed-effects panel data estimation, ensuring the validity of the results despite spatial dependencies in the data. It provides compelling evidence that both risk of poverty and income inequality are associated with poorer health outcomes, with risk of poverty having a stronger and more consistent effect. The regression results, Model 1, indicate that a one-unit increase in the risk of poverty (RPOV) is associated with a 2.113 unit increase in the Mental Health Index (MHI), a 2.071 unit increase in the Coronary Disease Index (CDI), a 1.218 unit increase in the Musculoskeletal Disorder Index (MDI), and an 11.92 unit increase in mortality (MOR). This aligns with existing research highlighting the detrimental impact of socioeconomic deprivation on mental well-being, including increased stress, anxiety, and vulnerability to mental health disorders. Model-I focuses on the effect of risk of poverty (RPOV) on health outcomes, while controlling for other relevant factors.

Model II examines the influence of income inequality (GINI) on health outcomes, again considering control variables. The regression results, Model 2, indicate that a one-unit increase in the Gini coefficient (GINI) is associated with a 0.469 unit increase in the Mental Health Index (MHI), a 0.516 unit increase in the Accidental Injury Index (AII), a 0.464 unit increase in the Musculoskeletal Disorder Index (MDI), a 0.281 unit increase in the Kela’s Mortality Index (KMI), a 0.375 unit increase in the THI’s Morbidity Index (TMI), and a 3.730 unit increase in Mortality (MOR). This suggests a potential link between wider income inequality and a higher prevalence of health issues. Model III shows the outcomes of the interaction effect of both RPOV and GINI. The interaction term between RPOV and GINI is positive and statistically significant, indicating that the negative impact of poverty on health outcomes is exacerbated by higher levels of inequality. This suggests that policies aimed at reducing both poverty and inequality may be particularly effective in improving health outcomes.

Table 4 employs a fixed-effect (FE) model to investigate the association between socioeconomic factors, including the risk of poverty (RPOV), and various health outcomes. It delves into the influence of RPOV, the primary explanatory variable of interest, on health outcomes, as presented in Model I. The analysis reveals that municipalities with a higher risk of poverty (RPOV) tend to experience poorer health across various dimensions. This is reflected in the positive and statistically significant coefficients for RPOV in most health outcomes. For instance, a higher RPOV is associated with a 1.40 point decrease in the MHI score. Similarly, a higher risk of poverty is linked to a 1.21 point increase in the CDI, 0.40 point increase in the CVDI, 0.32 point increase in MDI, 0.12 point increase in KMI, 0.28 point increase in KDI, 0.721 point increase in the TMI, and a 8.16 point increase in the MOR suggesting greater prevalence health issues among those facing poverty. These findings highlight the social determinants of health. Poverty likely creates stress, limits access to quality healthcare and healthy food, and restricts resources for preventive measures. This can lead to a cascade of negative effects, impacting both physical and mental health. The results underscore the importance of addressing poverty as a public health concern, considering the substantial impact it has on various health indicators, as evidenced by the coefficient values.

The model also incorporates various control variables to account for other factors that might influence health outcomes. For example, education (EDU) is generally associated with better health, as reflected by its negative coefficient. Conversely, unemployment (UN) shows mixed effects, potentially indicating the stress of unemployment or the support provided by social assistance (SA), which has a mostly positive association with health outcomes in this model. Family size (FAM) has a weak negative association with some health outcomes, while factors like education reform (EDR), migration (MIG), and medical expenditure (MEXP) have varying influences depending on the specific health outcome. By considering these control variables, the analysis strengthens the case for RPOV as a key factor influencing health.

Table 5 shows the connection between income inequality and health outcomes, using a fixed effect (FE) model. Model II focus on the main explanatory variable—income inequality (measured by the Gini coefficient)—and its influence on health outcomes. The analysis reveals a higher income inequality is associated with poorer health across various dimensions. This is reflected in the positive and statistically significant coefficients for Gini in most health outcomes. For instance, a one-unit increase in the Gini coefficient is associated with a 0.39 point decrease in the MHI score. This suggests that in societies with greater income inequality, people tend to experience worse mental health on average. Similarly, a higher Gini coefficient is linked to a 0.57 point increase in the AII, 0.21 point increase in the KMI, 0.30 point increase in TMI and a 2.06 point increase in the MOR. These findings point towards a potential increase in health issues with greater income inequality. Societies with a wider income gap may prioritize allocating resources towards public health initiatives for wealthier populations. This unequal distribution of resources could potentially have adverse effects on the health of the entire population, including individuals with higher incomes. Moreover, those in lower socioeconomic groups may experience chronic stress, encounter barriers to accessing quality healthcare and nutritious food, and face challenges affording preventive measures. This combination of factors can notably deteriorate both physical and mental well-being.

The model also considers other factors that might influence health. For example, education (EDU) is generally associated with better health outcomes, as reflected by its negative coefficients (often statistically significant). Conversely, the impact of unemployment (UN) is mixed, with potentially positive associations due to social assistance access, but also negative effects from joblessness stress. Similarly, other control variables like family size (FAM), education reform (EDR), migration (MIG), and medical expenditure (MEXP) show varying influences depending on the specific health outcome and significance level (coefficient values and *p*-values). By taking these factors into account, the analysis strengthens the case for income inequality (Gini) as a key factor influencing health.

Table 6 shows the connection between poverty, income inequality, and health outcomes. In Model III the key element is the interaction term between risk of poverty (RPOV) and income inequality (GINI) along with control variables. The analysis reveals that poverty itself is associated with poorer health across various dimensions. This is reflected in the positive and statistically significant coefficients for RPOV in most health outcomes including MHI, CVDI, MDI, KDI, and MOR scores suggesting worse health for those facing financial hardship. Income inequality, as measured by the GINI coefficient, also shows a concerning association with health. The positive and statistically significant coefficients for GINI suggest that societies with a wider income gap tend to have poorer health outcomes including including MHI, CVDI, MDI, and MOR. This might be because such societies allocate fewer resources towards public health initiatives, ultimately affecting everyone’s health. Additionally, individuals in lower socioeconomic groups may face chronic stress, limited access to quality healthcare and healthy food, and struggle to afford preventive measures. This combination of factors can significantly deteriorate both physical and mental well-being.

However, the most concerning finding emerges from the interaction term. This term captures how the impact of poverty on health changes depending on the level of income inequality. The positive and statistically significant coefficients for the interaction term paint a particularly bleak picture. In essence, while both poverty and income inequality have negative health effects, their combined influence is even more detrimental. Societies with a wider income gap may allocate fewer resources towards public health initiatives, further straining the health of those facing poverty. This can lead to a more significant burden of chronic diseases and potentially higher mortality rates for individuals in poverty residing in societies with greater income disparity. For example, a one-unit increase in the interaction term is associated with a 0.014 point increase in the MHI, 0.025 point increase in CDI, 0.020 point increase in CVDI, 0.116 point increase in MDI, 0.035 point increase in KDI, 0.025 point increase in TMI, and a 0.116 point increase in the MOR. Overall, this analysis highlights the critical need to address both poverty and income inequality to create a more equitable and healthy society. This can lead to a healthier population overall, with a reduced burden of chronic diseases and mortality, especially among those facing poverty.

The Granger causality test results, Table 7, reveal significant bidirectional causality between risk of poverty (RPOV) and the mental health index (MHI), suggesting a cyclical relationship where poverty exacerbates mental health issues, and deteriorating mental health increases poverty risks. Similarly, RPOV Granger-causes other health indices, such as cancer index (CI), coronary disease index (CDI), accidental injury index (AII), and cerebrovascular disease index (CVDI), indicating the strong influence of economic vulnerability on health outcomes. However, the reverse causality from health indices to RPOV is equally significant, highlighting how health burdens deepen poverty traps.

**Table 7:** Pairwise Grangar causality test

Null hypothesis:	F-statistic	Prob.
RPOV does not Granger Cause MHI	19.7038	3.E-09
MHI does not Granger Cause RPOV	42.6545	4.E-19
GINI does not Granger Cause MHI	0.74416	0.4752
MHI does not Granger Cause GINI	9.42235	8.E-05
RPOV does not Granger Cause CI	4.15161	0.0158
CI does not Granger Cause RPOV	46.8903	6.E-21
GINI does not Granger Cause CI	5.43388	0.0044
CI does not Granger Cause GINI	1.36511	0.2554
RPOV does not Granger Cause CDI	38.1938	3.E-17
CDI does not Granger Cause RPOV	220.505	5.E-94
GINI does not Granger Cause CDI	54.9790	2.E-24
CDI does not Granger Cause GINI	0.54869	0.5777
RPOV does not Granger Cause AII	15.7578	1.E-07
AII does not Granger Cause RPOV	36.1604	2.E-16
GINI does not Granger Cause AII	11.7947	8.E-06
AII does not Granger Cause GINI	4.00129	0.0183
RPOV does not Granger Cause CVDI	12.8131	3.E-06
CVDI does not Granger Cause RPOV	52.5257	2.E-23
GINI does not Granger Cause CVDI	9.77168	6.E-05
CVDI does not Granger Cause GINI	2.04231	0.1298
RPOV does not Granger Cause MDI	41.6074	1.E-18
MDI does not Granger Cause RPOV	383.617	3E-160
GINI does not Granger Cause MDI	2.03178	0.1312
MDI does not Granger Cause GINI	36.7182	1.E-16
RPOV does not Granger Cause KMI	2.31875	0.0985
KMI does not Granger Cause RPOV	97.0323	2.E-42
GINI does not Granger Cause KMI	1.02183	0.3600
KMI does not Granger Cause GINI	5.70964	0.0033
RPOV does not Granger Cause KDI	4.42442	0.0120
KDI does not Granger Cause RPOV	225.908	1.E-96
GINI does not Granger Cause KDI	0.59431	0.5520
KDI does not Granger Cause GINI	19.7644	3.E-09
RPOV does not Granger Cause TMI	31.6117	2.E-14
TMI does not Granger Cause RPOV	239.345	3E-102
GINI does not Granger Cause TMI	40.3341	4.E-18
TMI does not Granger Cause GINI	61.6760	2.E-27
RPOV does not Granger Cause MOR	81.1581	1.E-35
MOR does not Granger Cause RPOV	175.996	8.E-76
GINI does not Granger Cause MOR	4.70955	0.0090
MOR does not Granger Cause GINI	3.60534	0.0272

The variables RPOV, GINI, MHI, CI, CDI, AII, CVDI, MDI, KMI, KDI, TMI, and MOR represent Risk of Poverty, Gini Coefficient, Mental Health Index, Cancer Index, Coronary Disease Index, Accidental Injury Index, Cerebrovascular Disease Index, Musculoskeletal Disorder Index, Kela’s Mortality Index, Kela’s Disability Index, THL’s Morbidity Index, and Mortality, respectively

Income inequality (GINI) shows a weaker causal relationship with health indicators. GINI does not Granger-cause MHI, but MHI significantly Granger-causes GINI, implying that mental health disparities may contribute to income inequality through reduced labor productivity or increased social exclusion. Similarly, while GINI Granger-causes certain indices (e.g., CI, CDI, and TMI), the feedback effects from these health measures to inequality are not consistently significant. These results underscore the complex interplay between economic and health dynamics, advocating for multidimensional policies targeting both poverty alleviation and health improvements to break this cyclical interdependence.

## Conclusion

This study examined the relationship between poverty, income inequality, and health outcomes across Finnish municipalities from 1990 to 2023. The findings reveal that both poverty risk and income inequality adversely affect health outcomes, with wider income gaps correlating with poorer population health. This decline is likely driven by reduced public health investment and heightened stress among lower socioeconomic groups. Individuals in poverty face lower mental health scores and greater risks of chronic illnesses and mortality, highlighting the substantial health burden associated with economic deprivation. Furthermore, the interaction between poverty and inequality exacerbates these negative outcomes, influencing a wide range of health indicators, including cancer, coronary disease, accidental injuries, cerebrovascular conditions, musculoskeletal disorders, mortality rates, and overall morbidity.

Positive and statistically significant coefficients underscore the synergistic relationship between poverty and inequality, demonstrating how their combined impact amplifies health risks, particularly for vulnerable populations. These findings emphasize the interdependence of inequality and poverty in shaping health outcomes. High inequality intensifies the adverse effects of poverty, while low poverty mitigates some of the damage caused by inequality. This dynamic relationship underscores the need for nuanced policy interventions that address both factors in tandem, as the effects of inequality and poverty may vary across different geographical and social contexts.

The study is not without limitations. It employs a panel data design, limiting causal inference, and uses income-based poverty measures that offer only a partial picture of deprivation. Additionally, the absence of individual-level data restricts the ability to link economic deprivation directly to health system usage, raising concerns about ecological fallacy. Future research should adopt multilevel and longitudinal designs incorporating individual-level data, which would provide deeper insights into the interplay between economic and health factors and allow for more robust policy recommendations.

## Data Availability

Data used in this study are publicly available from the Finnish National Institute for Health and Welfare (THL) via the Sotkanet service: https://sotkanet.fi/sotkanet/en/index.

## Footnotes

1 For more detail see the report of the World Health Organization (WHO) established the Commission on Social Determinants of Health (CSDH) in 2005 that shows the importance of good health, significant disparities exist across populations. This commission aimed to support countries and global health partners in tackling the social roots of poor health and health disparities. Their primary focus was raising awareness about these social determinants and advocating for improved social conditions, particularly for vulnerable populations. The commission delivered its final report in 2008, outlining key recommendations such as improving living conditions, addressing unequal resource distribution, and promoting ongoing evaluation of interventions.
